# Ear Acupuncture Points in Neonates From Drug-Dependent Mothers: A Prospective Study

**DOI:** 10.3389/fped.2021.668248

**Published:** 2021-06-17

**Authors:** Kirsten Stähler van Amerongen, Annette Kuhn, Daniel Surbek, Mathias Nelle

**Affiliations:** ^1^Frauenklinik Inselspital, Department of Gynecology and Obstetrics, University hospital and University of Bern, Bern, Switzerland; ^2^Frauenklinik Inselspital, Division of Neonatology, University hospital and University of Bern, Bern, Switzerland

**Keywords:** acupuncture, ear-acupuncture point, neonates, drug-dependent mothers, Finnegan

## Abstract

**Aim:** The aim of the study was to determine the presence or absence of ear acupuncture points (EAP) in newborn children with or without neonatal abstinence syndrome (NAS) and to confirm the hypothesis that neonates with NAS have more EAP than healthy neonates.

**Methods:** We conducted a prospective case control study with ethical consent at the University Children's Hospital, Division of Neonatology Bern and the Department of Gynecology and Obstetrics Inselspital Bern in Switzerland. We determined the EAP in *n* = 26 newborn children born to drug-dependent mothers compared with *n* = 50 healthy newborns. For the detection of EAP, we used an ear point detection pen. EAP are present only if weakness exists in the corresponding area.

**Results:** Twenty-six neonates who were born to drug-dependent mothers and developed NAS were screened on the 5th day after delivery (range 1–22). The median Finnegan Score was 12 points (range 6–18) on the day of examination. Twenty-four active EAP were detected on the left earlobe and 25 were detected on the right earlobe. There was no significant difference between the right and left lobes (*p* = 0.9285, two tailed test) and the number of acupuncture points. The correlation between the Finnegan Score and the number of EAP was highly significant (*p* = 0.0001). The most common active points were the psycho-vegetative rim of the reflex zone of sympathicus and parasympathicus. Organic points were also commonly detected. The urinary bladder, kidney and hip points were detected with a frequency of 12–15%. The shen men pain point was found in three neonates, and the point of desire as a psychological point, was also detected. The correlation between sex and active EAP was highly significant (*p* = 0.0093, Mann-Whitney test for the left earlobe and *p* = 0.0025 for the right earlobe). Boys had a significantly higher number of EAP than girls. All NADA points were detected in the neonates born to drug-dependent mothers, and the most frequent point was the vegetative point. Healthy neonates showed only the vegetative point in the vegetative rim 1/3 among the NADA points. A comparison of newborns born to drug-dependent mothers and 50 healthy neonates showed that the former group had statistically significantly more active points. For the left earlobe, the difference between neonates born to drug-dependent mothers and controls was statistically significant (*p* = 0.0008, Mann-Whitney test). Highly similar results were found for the right earlobe (*p* = 0.0001, Mann-Whitney test).

**Discussion:** Our current work confirms that neonates born to drug-dependent mothers with high Finnegan scores and NAS have more EAP than healthy neonates. The vegetative rim is the most common point as shown in our previous studies. Our observations showed that twins had similar but not identical points; each individual had unique points depending on health status. Newborn boys with NAS had a higher number of EAP than newborn girls in the neonatal intensive care unit. This findings may be attributed to the reserve of newborns with NAS. Newborn girls are considered more robust than boys in the neonatal care setting. EAP in neonates might potentially be used for diagnosis and therapeutic opinions in neonates in the future.

## Introduction

The published literature on ear acupuncture mainly focuses on adults and children. Nogier (1), a French physician, introduced ear acupuncture in Marseille in 1957. In adults, ear acupuncture points (EAP) can be identified with a point detection device, which confirms acupuncture points on the basis of altered electrical conductivity (2). Those points can be used for diagnosis and therapy and are sensitive to pressure and palpation. They correspond to a body reflex area. In contrast to body acupuncture points, EAP are detectable only if they are irritated and are present only if a weakness exists in the corresponding area (2–4).

The current literature on EAP in neonates is scarce. We previously (5) demonstrated the presence of EAP in neonates and found that their presence is not dependent on sex, delivery mode or the affected earlobe on the right or left side (5). The psycho-vegetative rim was the most important point and an absence of psychic points in favor of organ points was observed. In a subsequent publication, we described an investigation of newborn triplets. The healthy girl of the triplets had only several EAP, and the two boys of the triplets with feto-fetal transfusion syndrome showed more points. The sickest child demonstrated the most EAP, and we observed a correlation between the numbers of points and the clinical state of health (6). Neonates with a medical problem had a significantly higher number of EAP than healthy neonates.

In this prospective case-control study, we aimed to examine newborn babies born to drug-dependent mothers, because these children are usually particularly ill. We applied standardized measurable methods using the Finnegan score to evaluate the health status of newborns with a measurable point system. We sought to examine a cohort of “diseased” newborns, according to the conventional medical definition, and to compare them with a healthy cohort. The children of drug-dependent mothers have withdrawal symptoms starting shortly after birth and lasting as long as several weeks. These children often need medication for their symptoms of withdrawal, which can be severe and may lead to seizures. Acupuncture is known to reduce symptoms of addiction in adults and alleviate withdrawal symptoms.

In recent years, parental requests for alternative non-invasive therapies have grown. Particularly in the United States complementary medicine is used for the treatment of chronic diseases (7, 8), and acupuncture is among the most frequently used therapies (9, 10). At present, ear acupuncture is mainly used for treating acute painful disorders, Yang diseases and drug addictions (3). Pomeranz (10) has demonstrated the influence of endorphin synthesis through acupuncture. The presence of endorphins is a likely explanation of how and why acupuncture works (10). Some studies have demonstrated this effect of less pain in children (11, 12), and animal studies have shown a positive effect of acupuncture on pain (13). Relative contraindications for acupuncture are pain of unknown origin; cancerous or infectious diseases or current medication with sedatives, neuroleptics or drugs similar to morphine. Additional contraindications are life threatening diseases or acute inflammation of the earlobe itself (3).

In 1972 and 1973, Weng HL from Hong Kong and Cui from the Neuroscience Institute of Beijing described the first study of acupuncture's effects on opiate withdrawal in addiction (14). In 1985, Smith (15), head of the National Acupuncture Detoxification Association (NADA) in New York, United States, used only five points on the ear to treat drug abuse. In Germany, Raben (16, 17) described acupuncture using the NADA points to treat both non-pregnant and pregnant drug-dependent women. In 2019, Litscher (15) summarized the main scientific findings from 27 publications describing ear acupuncture according to NADA.

Neonatal abstinence syndrome (NAS) has dramatically increased over the past decade. Acupuncture has demonstrated efficacy in adults experiencing withdrawal from addiction. Jackson (18), according to Preferred Reporting Items for Systematic Reviews and Meta-Analyses (PRISMA) guidelines, has reviewed *n* = 401 publications on acupuncture as a potential intervention for neonatal abstinence syndrome and found that acupuncture appears to be a safe and effective method for reducing withdrawal symptoms in infants with NAS.

The objective of our study was to detect EAP in newborns born to drug-dependent mothers compared with a control group of healthy neonates. Specifically, we aimed to determine the presence and absence of EAP in neonates with and without NAS.

## Patients and Methods

### Study Design

We conducted a prospective case-control study with ethical consent at the University Children's Hospital, Division of Neonatology Bern and the Department of Gynecology and Obstetrics Inselspital Bern in Switzerland. Examinations were performed between September 2002 and March 2005. The local ethics committee approved the study (KEK Nr. 142/02). Written informed consent was obtained from the mothers.

### Study Population

Eligible patients in the study group were newborns born to drug-dependent mothers who provided written consent to the examination. All included neonates were born at the Department of Obstetrics and Gynecology and direct consultation was provided by the Division of Neonatology in the same hospital for drug-dependence during pregnancy.

Patients whose mothers were unable to communicate in one of Switzerland's national languages (German, French, or Italian) or English, or mothers who refused to consent to the examination were excluded.

### Study Intervention

The drug-dependent mothers were in an interdisciplinary care setting during pregnancy, childbirth, and puerperium. Infection status testing was available for mothers.

Mothers in the study group were enrolled in a drug substitution program, thus indicating their high motivation for drug abstinence. In addition, the mothers were highly interested in their children's welfare and giving them relief from NAS.

We prospectively examined *n* = 26 newborn children born to drug-dependent mothers and determined their EAP.

The examinations for EAP were performed on the 5th day post-partum by the principal investigator, a certified physician acupuncture specialist. The acupuncture point search was performed only once on the right ear and only once on the left ear.

Each newborn's clinical state was defined by the Finnegan score, which was examined three times daily by a neonatologist. The specialized doctors and nursing staff in the neonatology department were trained personnel, thus ensuring the reliability of the Finnegan score. The Finnegan score consists of clinical standardized parameters to assess newborns with NAS (crying, sleeping after feeding, Moro reflex, tremor at rest or after stimulation, muscle tone, myoclonus, epileptic fits, sweating, body temperature, yawing, sneezing, blocked nose, breathing, tachypnea, feeding, and stool consistency). The routine daily clinical Finnegan score continued to be assessed independently of the treating clinical team. The values were distributed on a scale from a minimum of 0 to a maximum of 42. For Neonates with a score >11, opioid therapy was considered. When the score was <9, reduction in therapy was considered. The Finnegan score was determined at the same day and time of the EAP examination.

We recorded the delivery mode, sex, gestational age, birth weight, lengths at birth, Apgar score, umbilical arterial and venous pH, weight at the examination date, and presence or absence of NAS by using the Finnegan score. Term deliveries were defined as those after 37 completed weeks of gestation, and preterm deliveries were defined as those before 37 weeks of gestation.

From the mothers, we recorded age, parity and a voluntary statement of drug consumption. All pregnant women were under surveillance in a special drug substitution program for drug dependent women at the University Hospital of Bern. Screenings results for hepatitis B and C, and HIV were documented in the maternal medical record as part of pregnancy care.

For detection of EAP, we used a SVESA 1070 (Neuralstift Svesa 1070, Muenchen, Germany) CE certified ear point detection pen, an electrical device placed loosely on the outer surface of the ear. The SVESA neural pen is held in the examiner's hand like a writing pen. By placing the elongated search tip flat against the earlobe of the ear to be examined, the device is first calibrated to the skin condition, e.g., moisture. By placing the thin tip of the pen vertically on the surface of the skin, the ear can be systematically scanned. The illumination by the red light-emitting diode on the neural pen then signals whether the point is pathological. The electrical impedance conversion at a region with altered skin resistance is visualized *via* the red light-emitting diode, thus enabling detection of auricular acupuncture points by measuring the electrical skin resistance with a neural pen. This pen indicates the presence of an EAP through an optical signal than corresponds to diminished electrical skin resistance.

The right earlobe and then the left ear were tested according to a standardized examination procedure for EAP. Each neonate underwent only one examination only. Classification of active EAP was performed with a schematic ear graph according to the French and Chinese system from Hecker (19). Acupuncture points are described according to this international nomenclature. The acupuncture specialist was responsible for the assignment of EAP (KSvA).

Additionally, we searched for NADA points, which are often used for the treatment of drug addiction according to the NADA protocol (20). The NADA points are five EAP representing vegetative, shen men, kidney, liver, and lung points.

Third, we compared the data collected by our study group with the results of a control group from our previous study (5).

### Study Outcome

The primary outcome was detection of the type and number of EAP from the right and left earlobes in newborn children born to drug-dependent mothers. We aimed to identify which points were detectable on the right or left earlobe for girls and boys. The secondary outcome was analysis of the presence of NADA points (vegetativum, shen men, kidney, liver, and lung) in newborns born to drug-dependent mothers. The third outcome was comparison of the presence of EAP in newborns born to drug-dependent mothers with those in the healthy neonate group.

### Statistical Analysis

Statistical analysis was performed by a professional statistician using a stby Stat Xact version 6.0 from Cytel Studio. Graph Pad Stat Mate 2 version 4.0 for Windows was used for power analysis, with a power of 80%, an alpha of 0.05 in a two tailed test and a δ of 1.10. For comparison of the study population to controls with a power of 80%, an alpha value of 0.05 and a δ of 1.38, we required 25 matched pairs. Healthy mothers using matched pairs. Two-sided comparisons between healthy neonates and those born to drug-dependent mothers were performed with the exact Mann-Whitney U test. A *p*-value of 0.05 was considered significant at the 5% level.

## Results

### Study Population

The age of the mothers at the time of delivery was 30.5 years (median, range 21–40 years). Of those, 9 (34.6%) were primiparae, and 17 (65.4%) were multiparae.

Analysis of the infection status of the mothers showed positive for hepatitis B in 46% (*n* = 12), hepatitis C in 46% (*n* = 12) and hepatitis B+C+HIV in 4% (*n* = 1). Thirteen of the mothers (50%) were negative for hepatitis B and hepatitis C, and 20 were HIV negative (77%). In six cases, the data were incomplete. Data were obtained from the medical history completed by the mothers.

The analysis of substance abuse showed that methadone was the most frequent drug (69.2%), followed by heroin (50%), cocaine (31%) and cannabis (11.5%), benzodiazepine (11.5%), and marijuana THC (11.5%). All mothers had occasional nicotine and alcohol abuse and 73% were dependent on multiple substances. Methadone was used at a median of 52.5 mg/day (range 0–140 mg). Heroin consumption varied between 400 mg and 5 g daily (median).

Five of the neonates examined were preterm deliveries (19.2%) and 21 were term deliveries (80.7%). There were 10 normal vaginal deliveries (38.5%), two vacuum extractions (7.7%) and 14 deliveries *via* cesarean section (53.9%). Indications for delivery included pathological Doppler and growth restriction, fetal distress (CTG), placental abruptions, mal-positioning (breech presentation), failure to progress and maternal indications such as cardiac problems. The median birthweight was 2,683 g (range 1,260–4,290 g).

All neonates had been generally examined by an experienced neonatologist to evaluate the cardiovascular system and neurological status within the 1st 24 h after delivery.

All participants in the control group *n* = 50 were healthy neonates from our previous study (5). They did not undergo toxicology testing.

The demographic data of the newborn children are shown in Table 1.

**Table 1:** Shows the demographic data of the study group and the control group.

**Characteristics**	**Newborns of** **drug abusing mothers** ***n* = 26**	**Control group** ***n* = 50**
Mode of delivery	*n* = 10 vaginal delivery 38.5%	*n* = 25 vaginal delivery 50%
	*n* = 2 vacuum extraction 7.7%	*n* = 4 vacuum extraction 8%
	*n* = 14 cesarean section 53.9%	*n* = 21 cesarean section 42%
Sex	*n* = 16 male 61.5%	*n* = 27 male 54 %
	*n* = 10 female 38.5%	*n* = 23 female 46 %
Gestational age (weeks)	38 SSW ± 2.66	39 SSW ± 4.29
	(32 – 41 6/7 SSW)	(26 2/7 – 41 2/7 SSW)
Preterm delivery <37 weeks	*n* = 5 19.23%	*n* = 19 38%
Weight at birth (grams)	2,683 g ± 661.42	2,990 g ± 841.18
	(1,260 – 4,290 g)	(800 – 4,680 g)
<2,500 g	*n* = 9 34.62%	*n* = 16 32%
Length (cm)	47 cm ± 3.65	49 cm ± 4.63
	(38 – 53 cm)	(32 – 52 cm)
Umbilical arterial pH	7.28 ±0.07	7.29 ± 0.08
	(6.96 – 7.35)	(6.98 – 7.39)
Umbilical venous pH	7.34 ± 0.07	7.37 ± 0.08
	(7.04 – 7.4)	(7.05 – 7.51)
1 min Apgar-score	8 ± 1.72	8 ± 1.67
	(2 – 9)	(3 – 9)
5 min Apgar-score	9 ± 1.01	9 ± 0.67
	(5 – 10)	(7 – 10)
10 min Apgar-score	9 ± 0.56	9 ± 0.57
	(8 – 10)	(8 – 10)
Finnegan Score	12 range (6–18)	

*Values are noted as percent, median, standard deviation and range in brackets*.

### Results of the Study

The examination for detection of EAP on the ear surface was performed on the 5th day after delivery (range 1–22). Determination of the EAP was performed without difficulty by the acupuncture specialist.

#### Primary Results on the Type and Number of EAP on the Right and Left Earlobes, in Male and Female Neonates Born to Drug-Dependent Mothers

Twenty-four active EAP were detected on the left ear, and 25 on the right ear. In nine neonates (35%), no points on the left ear were found, and in six neonates (23%) no points on the right ear were found. Eleven neonates had identical points on the right and left ears. There was no significant difference between the right and left earlobe (*p* = 0.9285, two tailed test) and the number of acupuncture points.

The most common active point was the psycho-vegetative rim, the reflex zone of sympathicus, and parasympathicus. Organic points, e.g., the bladder, kidney, mouth, stomach, liver, lung and brain, were also detected.

The urinary bladder, kidney and hip points were detected with a high frequency of 12–15%.

The shen men pain point was found in three neonates, and the point of desire, as a psychological point, was also frequently detected (12%). Table 2 shows details of the type and frequency of the EAP found on the right and left earlobe in neonates born to drug-dependent mothers.

**Table 2:** EAP and NADA points in green on the right and on the left ear lobe of newborns from drug-dependent mothers.

	**Points left ear** ***n***	**%**	**Points right ear** ***n***	**%**
No points	9	35	6	23
Vegetative rim 1/3, Veg I	1	4	3	12
Vegetative rim 2/3	10	38	12	46
Vegetative rim 3/3	12	46	11	42
Urinary bladder point	3	12	4	15
Kidney	3	12	3	12
Mouth esophagus point	2	8	0	0
Esophagus	3	12	2	8
Cardia	2	8	1	4
Stomach	1	4	2	8
Duodenum	0	0	1	4
Jejunum / ileum	0	0	1	4
Liver	1	4	0	0
Lung	1	4	1	4
Endocrine	2	8	1	4
TSH	1	4	1	4
Hip joint	2	8	4	15
ISG	0	0	1	4
Thoracic vertebrae point	0	0	1	4
Thalamus point	2	8	1	4
Brain	1	4	0	0
Omega point II	2	8	2	8
Vegetative point I	2	8	2	8
Interferon point	2	8	2	8
Allergy point	2	8	1	4
Darwin point	2	8	2	8
Shen men	1	4	2	8
Point zero	1	4	0	0
Point of desire	3	12	3	12

**Figure 1:**
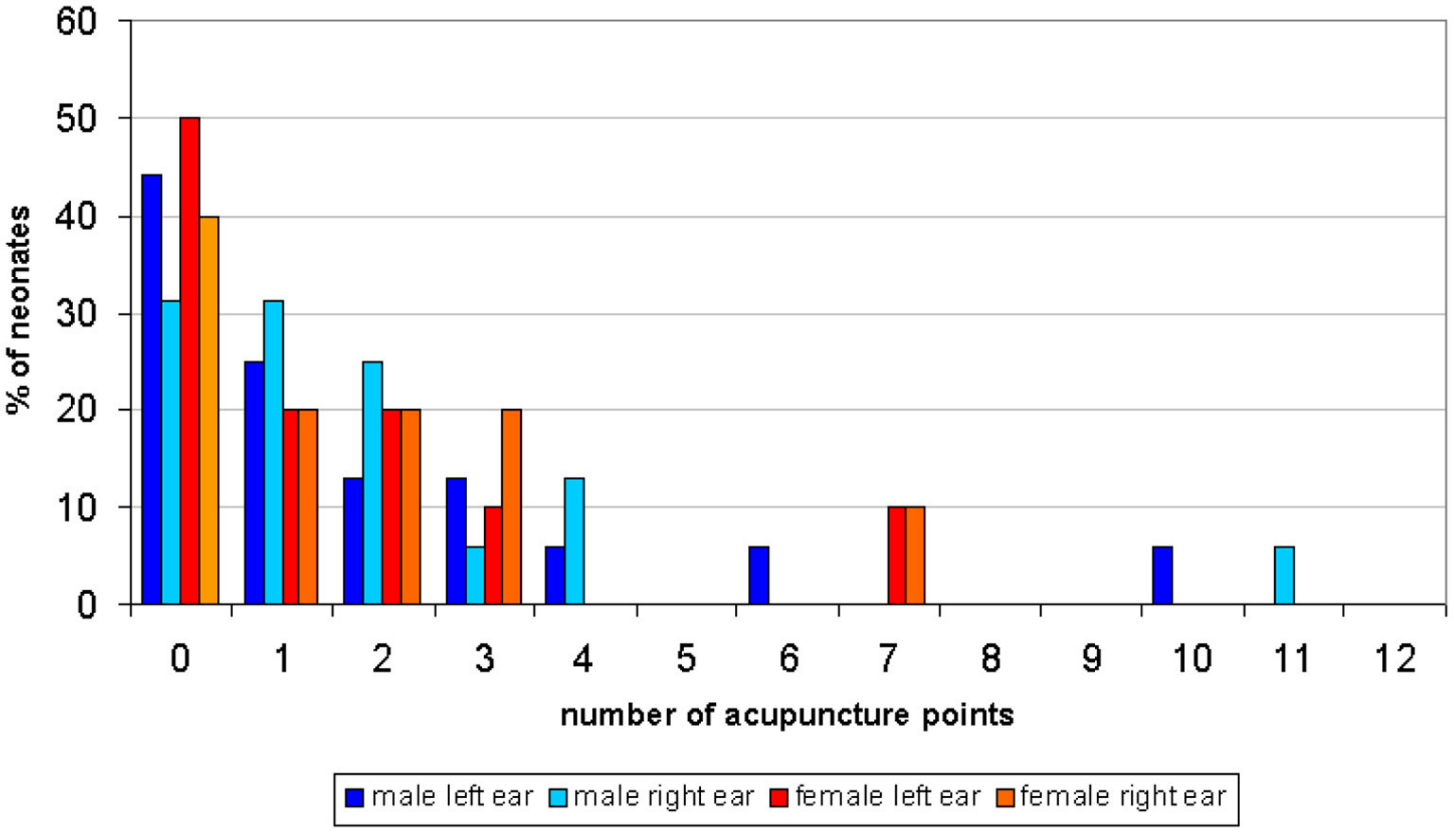
Demonstrates the number of active ear points in comparison to male and female neonates.

The correlation between the sex of the neonates and active EAP was highly significant (*p* = 0.0093, Mann-Whitney test for the left earlobe and *p* = 0.0025 for the right earlobe). Boys showed a significantly higher number of active EAP‘s than girls. Figure 1 shows the numbers of active ear points on the right and left earlobe in boys and girls.

#### Secondary Results Related to the Existence of NADA Points in Neonates Born to Drug-Dependent Mothers vs. Healthy Neonates

All NADA points were found in the neonates born to drug-dependent mothers. In Table 2, the NADA points and frequency are marked in green. The vegetative point in the original NADA concept comprised only one point. The neonates showed an entire area under the helix of the auricle, and thus the vegetative point was contained in the area of the vegetative rim 1/3.

Healthy neonates from our previous study showed only the vegetative rim 1/3 among the NADA points.

#### Tertiary Result of the Comparison of Healthy Neonates vs. Neonates Born to Drug-Dependent Mothers

The offspring of drug-dependent mothers had statistically significantly more active EAP than controls.

The difference between the active EAP on the left earlobe in neonates born to drug-dependent mothers compared with controls was statistically significant (*p* = 0.0008, Mann-Whitney test). Highly similar results were found for the right earlobe (*p* = 0.0001, Mann-Whitney test).

All neonates born to drug-dependent mothers developed NAS. Manifestations of NAS were observed at a median of 1st day after delivery (range 1–4 days). The median Finnegan score was 12 points (range 6–18). The length of hospital stay in the neonatal intensive care unit had a median of 46.5 days (range 23–95).

The correlation between the Finnegan score and the number of EAP was highly significant (*p* = 0.0001). This correlation was the same between girls and boys and between the right and left earlobe.

We observed that a newborn with a high Finnegan score of 14 points and clinical seizures showed a large number of active EAP (vegetative rim 1/3, 2/3, 3/3, urinary bladder, kidney, esophagus, endocrine rim, TSH, thalamus, omega point, interferon, and point of desire) during the examination.

The group of newborns born to drug-dependent mothers also included a dichorial-diamniote twin pregnancy born at the 34.4/7th week of pregnancy. The boy weighed 1,605 g and had an Apgar score of 8 9 9 at birth. The examination for EAP showed no points on the right or left earlobes. The girl had a birth weight of 1,965 g and an Apgar score of 7 8 9, and showed two active points during the examination (vegetative rim 1/3 and 2/3).

## Discussion

The **strengths** of the study include its prospective case-control design comparing study and control groups.

Our study presents a new potential method for assessing withdrawal in NAS. Newborns cannot verbally communicate where and what causes them pain or where problems lie in the harmony of the body. This search method for finding “problem areas” through EAP is a simple, inexpensive, non-invasive and quick method. If a correct finding of EAP or disturbed areas in the body enables the assignment to the corresponding clinical symptoms, this method could be used not only for diagnosis, but also for therapy in the future. Another strength is the interdisciplinary interaction between conventional medicine and complementary medicine.

Finding a disturbed area, e.g., kidney point through this complementary acupuncture search medical method could lead to further conventional medical diagnostics in the kidney area in children. In the future, the corresponding region in the body (e.g., the kidney) could be treated at the reflex zone on the ear (e.g., kidney point). Such a treatment would be interesting, e.g., for the shen men pain point. In my meaning–if pain could be identified in a child simply by palpating the auricle for EAP and subsequently treated with acupuncture, further treatment opportunities might be possible.

The **limitations** of the study included that the work was conducted more than a decade ago, although it remains relevant. Owing to the author's limited time resources in the past, publication was delayed. The motivation to publish the study recently increased, owing to the need for u-to-date results because of current trends.

Another study limitation is that the control group of healthy newborns was from our previous study in which no toxicology testing was performed. As many as 5–10% of pregnancies may involve substance- exposure, particularly to nicotine or alcohol.

The current study demonstrated the presence of significantly more active EAP in neonates born to drug-dependent mothers than in control neonates born to healthy mothers, on the basis of a matched pair design.

The results are consistent with our conclusion that neonates with medical problems such as NAS have more EAP than healthy newborns; they additionally confirm our previous findings in triplets with feto-fetal transfusion syndrome (6).

The observations of the newborns in our study with high Finnegan scores and high numbers of EAP confirm our thesis that newborns with medical problems show high numbers of active EAP.

The observation of twins in our study indicated that twins have similar but not identical points, each individual has unique points, depending on health status.

An Austrian group led by Raith has performed various studies on neonates; in which the psycho-vegetative rim was most common organic area in the children, followed by several organic points in neonates with NAS (21). However, Kurath-Koller (22) has found, not only organic, but also psychological points in newborns with NAS. The psycho-vegetative rim was the most common active somatic area in infants with NAS, followed by several somatic and psychic EAP. The most frequently found psychic points were the frustration point and the R point (22).

Our study confirms these findings and has produced the same results; however, we used a SVESA neural pen, whereas the Austrian studies used a Silverbauer neural pen. These findings confirm that the point search on the ear is a suitable method, because similar or identical results were achieved regardless of the examiner. Therefore, the results indicate the sensitivity and specificity, and the positive and negative value, of using the pen, thus supporting continued use of this method in the future.

Stadler and Raith (23) have also found that sick neonates have a significantly higher number of active EAP than and healthy neonates. They have shown that auricular medicine is an effective non-pharmacological approach for the treatment of pain in newborn infants to address symptoms of NAS (24). Raith et al. (25) conducted a prospective, randomized, controlled, blinded, single-center study between 2009 and 2014, treating neonates with NAS by using laser needles according to the NADA protocol in combination with pharmacological therapy, and compared the cases group to a control group.

Our current work confirms that neonates with high Finnegan scores have more active points than healthy neonates, and that the vegetative rim is the most commonly found point.

We observed a significant difference in the number of points found according to the sex of the neonates. Newborn boys with NAS had a higher number of active EAP. This finding may be attributed to the reserve of sick newborns. Newborn girls are considered more robust than boys in the neonatal care setting.

We recommend that the neural pen be used in a standardized manner, because some experience is required in applying the pen to newborn ears where the cartilage remains smooth. The examiner should carefully scan the small, soft ear of the newborn with the point selector and pay attention to the child's movement to avoid accidental injury. In our study, all examinations were performed by the same investigator, who had long-standing experience in using the neural pen on newborns before this study was conducted. Unfortunately, scientific data on the sensitivity, specificity and positive or negative predictive value of the pen are lacking.

The Austrian group has used an electrical point searcher device (PS3, Silberbauer, Vienna, Austria) for similar examinations on newborns. They pens differ in the signal display of the points found: the Svesa search pen shows an optical light signal, whereas the Silberbauer pen shows an optical and additional acoustic signal at an identified point. We believe thatxposed to as few stimuli as possible during the examination. The search with a point selector with a visible light signal is objectively visible and reproducible. However, the point search method by pulse measurement RAC is noticeable to only the investigator during the examination.

In adults, EAP has been clinically implemented for the treatment of drug-dependent patients (26, 27). Our study highlights the potential benefits in diagnostics and future treatment of drug-dependent infants. Raith's study has reported the first results of treatment with a non-invasive laser (28).

A potential problem in comparing studies using EAP is the variation descriptions and nomenclature in the currently used EAP charts (2, 3, 26–28). We used the French and Chinese nomenclature in our study and we believe that uniform use of EAP charts should be considered in the future, to ensure the comparability of study results. Although searching for points with the pen is reproducible, the assignment of the points differs slightly depending on the map and the experience of the examiner, thus representing a drawback of this method.

The measured points indicate the current condition of the newborn at the exact time of examination. If the neonates were examined at a point in time when severe disharmonies or medical problems were present, many points would be identified. If the same newborn were examined at a point in time when harmony or no disturbance in the body was present, then only few points would be identified.

If we consider the presence of EAP as indicative of a potential health hazard or weakness, we can speculate how these points were generated.

Antenatally, the offspring of drug-dependent mothers are exposed to various health threatening factors including not only the drugs themselves, but also malnutrition and infectious diseases such as hepatitis, HIV and sexually transmitted infections.

The details pertaining to drug consumption were recorded by self -administered questionnaires answered by the expectant mothers. Therefore, we cannot be certain that the mothers provided a correct list of all substances that they used. However, the mothers were encouraged to be honest and to list all substances to give their children the best postnatal support possible. The birth weights of neonates born to drug-dependent mothers were significantly lower than those of neonates not exposed to drugs in pregnancy, this findings is partly attributable to malnutrition in drug users.

In neonates, drug withdrawal symptoms usually appear during the 1st 24 h after birth and symptoms increase in the following days. The higher the drug dosage, the more severe the neonatal symptoms. Offspring of methadone users often have delayed withdrawal symptoms, usually starting 48 h post-partum (29). Because methadone has a longer half-life than heroin, the withdrawal symptoms appear later than those in heroin users (30–32). Late manifestation is particularly common in drug-dependent patients using multiple substances such as benzodiazepines and barbiturates, and may occur even after several weeks post-partum.

These findings corresponds to our experience in this study; the first signs of withdrawal manifested during the 1st 24 h after delivery and were treated with Tinctura Opii until the symptoms ceased.

Evaluation of neonatal withdrawal symptoms is performed with the Finnigan score (33, 34). Because we examined the EAP in neonates only once, we correlated the Finnigan score only at the specific time when the EAP were measured.

All neonates born to drug-dependent mothers developed NAS. In future studies, a longitudinal correlation of Finnegan score and EAP will be examined.

For women using heroin, methadone substitution decreases the necessity of drug substitution for newborn and avoids extreme fluctuations in maternal opioid levels.

Overall, methadone substitution results in higher birth weights and a positive maternal social impact (35–37), this issue is important for methadone substitution.

NADA is an American association that supports use of acupuncture for drug withdrawal in adults. Under the NADA concept, drug addicts are treated “vegetativum, shen men, kidney, liver, and lung” EAP, thus resulting in positive effects (15).

We also found that the most common active point was the psycho-vegetative rim. Additionally, organic points were detected. The shen men pain point, which is an indication of pain, was found in three children.

NADA points were also detected in newborns born to drug-dependent mothers and could potentially be used for the treatment of NAS in the future. A prospective randomized controlled study is planned to investigate this hypothesis. The current study showed significantly more EAP in the offspring of drug-dependent mothers than in healthy offspring.

We previously demonstrated the presence of EAP in healthy neonates and observed no dependence on sex, delivery mode or the affected earlobe (5). In 66% of neonates, no points were found. The psycho-vegetative rim was the most important point, and an absence of psychic points was observed in favor to organ points. No significant differences were found between the right and left earlobes, male and female neonates, or term and preterm deliveries. Moreover, there were no differences dependent on the mode of delivery.

When searching the literature, only English-language publications were considered. Essential findings or studies may not have been considered, for example, those published in Chinese.

## Conclusion

Neonates born to drug-dependent mothers had significantly more active EAP than those of healthy mothers. The correlation of EAP with the Finnegan score was highly significant. The most common active EAP were in the psycho-vegetative rim. Organic points, psychological points and NADA points were also commonly detected. Male neonates had a significantly higher number of EAP than female neonates.

## Data Availability Statement

The original contributions presented in the study are included in the article/supplementary material, further inquiries can be directed to the corresponding author.

## Ethics Statement

The studies involving human participants were reviewed and approved by local ethics committee of the canton Bern, Switzerland. Written informed consent to participate in this study was provided by the participants' legal guardian/next of kin.

## Author Contributions

KS and MN conceived the study and participated in operationalizing procedures of recruitment of the patients. AK participated in designing the study and calculated the statistical consideration. KS and AK wrote the ethics proposal and wrote the first draft of the manuscript. DS and MN supervised the research team and had the responsibility for obstetrics (DS) and neonatology (MN). KS have done the project coordination and the study examinations. All authors participated in the revision of subsequent drafts and approved the final version of the manuscript.

## Conflict of Interest

The authors declare that the research was conducted in the absence of any commercial or financial relationships that could be construed as a potential conflict of interest.

## Abbreviations

EAP: ear acupuncture points
NAS: neonatal abstinence syndrome
NADA: national acupuncture detoxification association.
